# 2541. Steady-state PK of Fixed Dose Dolutegravir+Rilpivirine in Hemodialysis

**DOI:** 10.1093/ofid/ofad500.2158

**Published:** 2023-11-27

**Authors:** Samir K Gupta, Allon Friedman, Desta Zeruesenay

**Affiliations:** Indiana University School of Medicine, Indianapolis, Indiana; Indiana University School of Medicine, Indianapolis, Indiana; Indiana University School of Medicine, Indianapolis, Indiana

## Abstract

**Background:**

Fixed dose combination (FDC) dolutegravir (DTG) plus rilpivirine (RPV) is an approved antiretroviral treatment regimen for people with HIV. The steady-state PK of FDC DTG+RPV in those requiring hemodialysis (HD) has not been previously studied.

**Methods:**

We performed a single-center, prospective evaluation of the steady-state PK of FDC DTG (50mg)+RPV(25mg) in HIV-negative adults either requiring HD (n=4; 2 men, 2 women) or with normal renal function, defined as CrCl ≥ 75mL/min (n=2; 1 man, 1 woman). All participants received DTG+RPV daily for 10-14 days with food before undergoing an intensive 24-hour PK evaluation (performed between dialysis days for those requiring HD). Plasma drug and metabolite concentrations were measured using a validated LC/MS/MS assay method (QTRAP 6500+LC-MS/MS system) with turboelectrospray source operating in both positive (confirmation) and negative (quantification) modes. We did not evaluate dialysis extraction of DTG+RPV. Descriptive PK parameters were calculated.

**Results:**

No participant experienced serious or grade 3-4 adverse events; there were no study discontinuations. The 4 HD and 2 normal renal function participants were of similar ages (range, 50-60 vs 53-58 years) and BMI (range, 18.5-28.2 vs 20.3-24.0 kg/m^2^). The Table shows the PK parameters assessed in the study population for circulating plasma DTG, DTG-glucuronide (DTG’s primary metabolite), and RPV.

**Figure:**
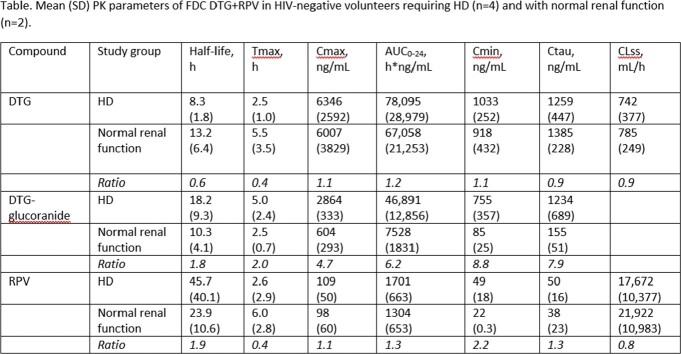

**Conclusion:**

In this study, HD did not lead to clinically appreciable differential exposures to DTG and RPV; the markedly increased exposure to DTG-glucoranide (which is considered inert) in HD suggests increased UGT1A1 activation. All participants maintained exposures throughout the dosing interval greater than the reported IC_90_ efficacy values for DTG (64ng/mL) and RPV (12ng/mL). These data suggest no dosing modifications are needed for the FDC DTG+RPV regimen in HD.

**Disclosures:**

**Samir K. Gupta, MD**, Gilead Sciences: Advisor/Consultant|ViiV Healthcare: Advisor/Consultant|ViiV Healthcare: Grant/Research Support **Allon Friedman, MD**, Eli Lilly: Advisor/Consultant|Eli Lilly: Stocks/Bonds|GI Dynamics: Advisor/Consultant|Gila Therapeutics: Advisor/Consultant

